# Opportunistic Interference Alignment for Spectrum Sharing between Radar and Communication Systems

**DOI:** 10.3390/s20174868

**Published:** 2020-08-28

**Authors:** Dong-Hwan Kim, Janghyuk Youn, Bang Chul Jung

**Affiliations:** 1 Department of Electronics Engineering, Chungnam National University, Daejeon 34134, Korea; dhkim0812@gmail.com (D.-H.K.); jhyoon@o.cnu.ac.kr (J.Y.); 2 Aircraft Radar System PMO, Agency for Defense Development, Dajeon 34186, Korea

**Keywords:** MIMO radar, MIMO communication, opportunistic interference alignment, spectrum sharing, transmit beamforming

## Abstract

In this paper, we propose a novel opportunistic interference alignment technique for spectrum-shared radar and uplink cellular communication systems where both systems are equipped with multiple antennas. In the proposed OIA technique, the radar system sends its signal so that the radar signal is received into interference space at base stations (BSs) of the cellular system, while each uplink user (UE) generates its transmit beamforming vector so that communication signals are received within interference space at the radar receiver. Moreover, to achieve better sum-rate performance of the cellular communication system, the BS selects the UEs which results in sufficiently small interference to other cells for the uplink communication. With the proposed OIA technique, detection performance of the radar system is protected, while the communication system achieves satisfactory sum-rate performance. Through extensive computer simulations, we show that the performances of both radar and communication systems with the proposed technique significantly outperform a conventional null-space projection based spectrum sharing scheme.

## 1. Introduction

Rapid developments of wireless communication technology and a consequent surge in the number of wireless devices using radio frequency have induced shortage of radio spectrum. Thus, spectrum management techniques for efficiently reusing underutilized radio spectrum have received much attention from both industry and academia in the last two decades [1,2,3,4,5,6,7,8,9,10,11,12,13,14,15,16]. In particular, U. S. spectrum regulatory agencies, i.e., the federal communications commission (FCC) and the national telecommunications and information administration (NTIA), has announced that the spectrum assigned to government agencies are significantly underutilized even in urban areas. On the other hand, the spectrum band used by commercial operators are being heavily utilized, which causes a lot of demand for the government agencies including military to share the spectrum band they are using with the commercial wireless systems [17,18].

However, while research on the spectrum sharing between wireless communication systems has been conducted very much, research on spectrum sharing between a radar system and a wireless communication system has not been conducted sufficiently despite the low utilization of the radio spectrum allocated to radar systems [19]. Different from spectrum sharing in communication-communication systems, in spectrum sharing in radar-communication systems, inter-system interference (ISI) has to be carefully managed because of sensitivity of radar and tremendously high transmit power of radar compared to communication system [20]. Recently, a few groups have drawn several research results on the spectrum sharing technologies between radar and communication systems [21,22,23,24,25,26]. The most challenging technical issue when both the radar and communication systems share the same spectrum band is obviously the inter-system interference to each other, which may significantly degrade the performance of both systems. Hence, in order for both systems to effectively share the same spectrum bands, such a harmful ISI needs to be mitigated or properly managed at least.

A null-space projection (NSP) with multiple antennas for the radar signal design was proposed to minimize the ISI from a military radar system to a cellular communication system [21,22,23,24]. Since only a single base station (BS) in the cellular system is allowed to share the same spectrum band with the radar system in [21,22], a BS selection algorithm was also proposed, where the BS that yields the minimum performance degradation at the radar system is selected for the spectrum-sharing. A cluster of BSs is selected for the spectrum sharing in [23] and a 3D channel model was adopted to consider direction of the BS from the radar system in [24]. The basic idea and methodology of NSP in [23,24] are the same as the original NSP in [21,22]. However, all BSs in cellular networks share the same spectrum band in practice, i.e., full frequency reuse, even though a single BS or a few BSs in the cellular network are assumed to share the same spectrum band with the radar system in [21,22,23,24]. Moreover, the reverse direction ISI from the cellular communication system to the radar system was also not considered in [21,22,23,24], even though the sensitivity of the radar system is very high and the wireless communication signal may deteriorate the radar detection performance severely [20]. In [25], an average ISI at the radar system from the cellular BSs was mathematically analyzed with exclusion zone, where cellular BSs are assumed to be equipped with a massive number of antennas and the locations of cellular BSs are modeled as a Poisson point process (PPP). In [26], a chance-constrained stochastic optimization technique was proposed to guarantee the minimum performance of the radar system, while maximizing the performance of cellular system. In particular, the transmit power adaptation at the cellular BSs is considered in [26]. Especially, addressing ISI with beamforming as [21,22,23,24] is considered in [27,28]. In [27], radar and communication systems are both optimized to maximize the probability of detection of radar while guaranteeing the transmit power budget of the BS and signal to interference plus noise ratio (SINR) of communication system. Moreover, same research group of [27] has proposed beamforming optimization based on the concept of constructive interference. Only BS beamforming is optimized to minimize transmit power while guaranteeing the received SINR at UE and interference threshold to radar or to minimize interference to radar subject to received SINR constraint. However, in both [27,28], only downlink communication scenario is considered even there is a probability that radar can be affected by uplink communication signal when radar is deployed nearby the UEs. It is worth noting that no ISI mitigation technique based on beamforming was proposed for the uplink communication system and radar coexisting environment.

Meanwhile, interference alignment (IA) techniques have received much interest as efficient multi-antenna based interference mitigation techniques in wireless communication systems [29,30]. The basic concept of IA is to confine interference from other users into a pre-defined linear space at the receiver at the user of interest and to separate the desired signal space from the interference space. In addition, an opportunistic interference alignment (OIA) technology has been proposed for effectively combining the IA technique with user scheduling technique for both multi-user downlink and uplink cellular networks [31,32,33,34,35,36,37,38]. The OIA technique opportunistically selects the users amongst all users in each cell in the sense that inter-cell interference (ICI) is aligned at a pre-defined interference space. It was shown that the OIA technique asymptotically achieves the optimal degrees-of-freedom if the number of users in each cell is large enough [32,33,36].

Recently, the IA techniques have been applied to effectively mitigate the ISI in the spectrum sharing radar and communication systems [39,40,41]. In [39], a joint pre-coder and post-coder design based on the IA principle was proposed for spectrum sharing between MIMO radar and MIMO communication systems and another joint transmit and receive beamformer design with a two-tier alternating optimization algorithm was proposed for spectrum shared MIMO radar and MIMO communication systems in [40]. An ergodic IA method for interference elimination in spectrum shared MIMO radar and multi-user MIMO communication systems was proposed in [41]. However, the conventional IA-based ISI mitigation techniques in [39,40,41] considered only *K*-user interference channel in communication systems even though most commercial wireless communication systems consist of BSs and multiple user equipments (UEs) which are belong to a certain BS. In addition, the OIA technique has not been applied to spectrum-shared MIMO radar and MIMO wireless communication systems so far in the literature.

In this paper, hence, we propose a novel OIA with radar (OIAR) for spectrum sharing between MIMO radar system and MIMO cellular uplink communication systems by considering not only the ISI from the radar system to communication system but also the ISI from the communication system to the radar system. In the proposed OIAR, communication system UEs generate transmit beamforming vector to minimize ISI to the radar system, while the radar system generates the beamforming matrix that will be used for eliminating the ISI from the radar to the BSs. Moreover, the UEs that minimize the other-cell interference are selected for uplink communication in each cell as in the conventional OIA framework. To validate the proposed OIAR technique, detection probability for the radar system and sum-rate for the communication system are evaluated through extensive computer simulations.

The rest of this paper is organized as follows. In Section 2, we describe system and channel models. In Section 3, we explain the overall procedure of the proposed OIAR technique and we introduce the performance metrics including the sum-rate for the communication system and the detection probability for the radar system in Section 4. The simulation results of the proposed OIAR are shown in Section 5. Finally, conclusion is drawn in Section 6.

## 2. System and Channel Models

**Figure 1:**
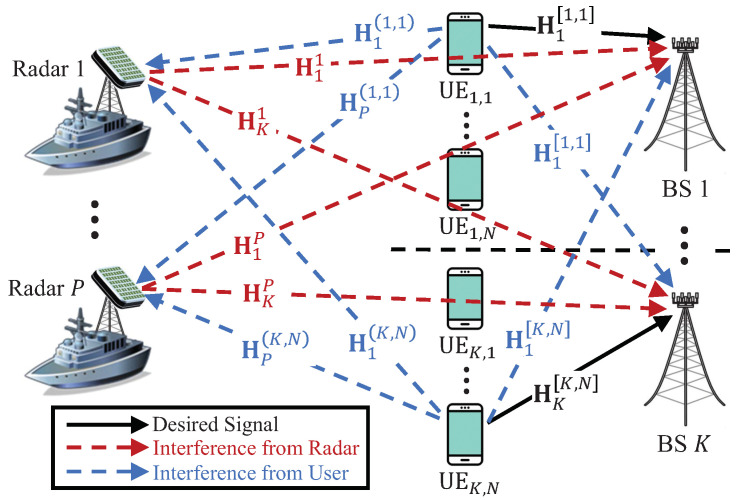
System model of spectrum-shared MIMO radar and MIMO communication systems.

First of all, please note that we consider the system that radar and communication system always share the spectrum. Hence, spectrum sensing operation is not needed but radar need to transmit reference signal (such as BS in communication system) so that UE acquire the interference channel between itself and radars to manage the ISI [27,41]. The system considered in this paper is shown in Figure 1. The radar system consists of *P* colocated MIMO radar transceivers equipped with *Y* uniform linear array antennas. Moreover, the communication system consists of *K* cellular communication BSs with *M* antennas, and *N* UEs with *L* antennas in each cell. In this paper, we assume that radar systems share the radio spectrum with uplink cellular networks, thus the radar signal transmission may interfere the BSs signal reception, and uplink UEs’ signal transmission may interfere the radar signal reception. In addition, all cells are assumed to operate over the same spectrum band, which implies full frequency reuse and inter-cell interference exists as well. We also assume that there is no interference among radars, since radar systems are carefully coordinated to guarantee performance and utilize a very sharp beamwidth in general [20]. In our considered system, only S(≤N) UEs in each cell are granted to transmit signal to their corresponding BS. Moreover, the number of selected UEs is smaller than the number of BS antennas (i.e., S≤M) to effectively mitigate intra-cell interference of each cell. Besides, the number of radar antennas is assumed to be larger than sum of the number of antennas of all BSs in the communication system (i.e., Y>KM), so that the radar systems reliably detect targets while does not induce any interference to communication systems [42]. Furthermore, a line-of-sight (LOS) between each radar and its target of interest is assumed.

The transmit steering vector and receive steering vector of the *p*-th radar are defined as

(1) $$\begin{array}{c} a_{p,t}=\left[1,e^{-i\pi \sin\left(\theta_{p,t}\right)},\cdots ,e^{-i\left(Y-1\right)\pi \sin\left(\theta_{p,t}\right)}\right],\;a_{p,r}=\left[1,e^{-i\pi \sin\left(\theta_{p,r}\right)},\cdots ,e^{-i\left(Y-1\right)\pi \sin\left(\theta_{p,r}\right)}\right], \end{array}$$

respectively, where the antenna spacing of array is assumed to be set as half of the wavelength. The phases $\theta_{p,t}$ and $\theta_{p,r}$ denote the direction of transmission and reception for the target of the *p*-th radar, respectively. Due to LOS condition between the radar and the corresponding target of interest, we can assume that $\theta_{p,t}=\theta_{p,r}=\theta_{p}$ and $a_{p,t}=a_{p,r}=a_{p}$. The channel coefficient matrix $H_{p}^{\left(i,j\right)}\in C^{Y\times L}$ denotes the interference channel between the *j*-th UE in the *i*-th cell and the *p*-th radar transceiver, and $H_{k}^{p}$ denotes the interference channel between the *p*-th radar and *k*-th BS. Furthermore, $H_{k}^{\left[i,j\right]}$ represents the communication channel between the *j*-th UE in the *i*-th cell and the *k*-th BS. We assume that all elements in matrices $H_{p}^{\left(i,j\right)}$, $H_{k}^{p}$, and $H_{k}^{\left[i,j\right]}$ are independent, identically distributed and complex Gaussian random variables with zero-mean and unit-variance. (Note that the radars are assumed to be located near the communication system in our system model and thus it is possible that the received signal-to-noise ratio (SNR) of the desired radar signal at radar systems has similar levels with interference-to-noise ratio (INR) of the interference signal from UEs of communication systems). We assume local channel station information (CSI) (i.e., CSI between itself and others) are available at all transmitting nodes in the system by the reference signals that is broadcasted from all receiving nodes as in [31,32,33,34,35,36,37,38].

## 3. Opportunistic Interference Alignment with Radar

Overall procedure of the proposed OIAR technique for the spectrum sharing radar and communication systems is explained in detail in this section. In the proposed technique, coordinated transmit and receive beamforming algorithms are exploited both in the radar and communication systems. To be specific, we first introduce how to separate the signal space and interference space for both the radar and the BSs reception as an initialization process, and then the transmit beamforming of the radar and the UEs are designed to minimize ISI and ICI. After beamforming design, how to select *S* UEs for uplink transmission in communication systems is explained.

### 3.1. Initialization: Separating Signal and Interference Spaces

**Figure 2:**
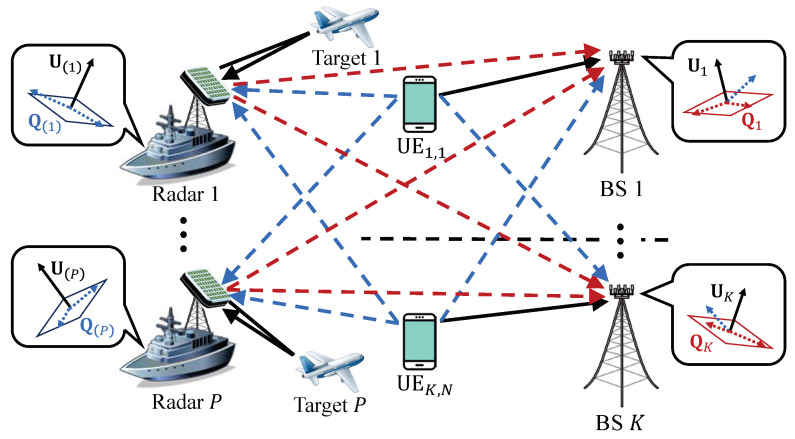
Signal space and interference space of radar and communication systems for OIA.

Figure 2 illustrates how to separate signal space and interference space at the radar and at the BS in the proposed OIAR technique. Since radars are interested only in detecting the desired signal from the target, the signal space of the *p*-th radar becomes the same as the space that generated by steering vector $a_{p}$. If we define a signal space for the *p*-th radar $U_{\left(p\right)}$ as $a_{p}$, then it can be written by

(2) $$\begin{array}{c} U_{\left(p\right)}=\frac{a_{p}}{∥a_{p}∥}. \end{array}$$

Note that the interference space of the *p*-th radar can be easily obtained by using the definition of null space of $U_{\left(p\right)}$. Similarly, the interference space of *k*-th BS is defined as $Q_{k}$, which is given by

(3) $$\begin{array}{c} Q_{k}=\left[q_{k,1},\cdots ,q_{k,M-S}\right]\in C^{M\times (M-S)}, \end{array}$$

where $q_{k,r}$ is a unit-norm vector. Then, the *k*-th BS calculates its signal space $\left(U_{k}\right)$ using $Q_{k}$ by the definition of null space as follows:

(4) $$\begin{array}{c} U_{k}=\mathrm{null}\left(Q_{k}\right)\in C^{M\times S}. \end{array}$$

After generating each space as addressed above, all BSs and all radars broadcast its signal space, then all UE acquire the effective channel between itself and every receiving nodes (i.e., radars and BSs) and all radar also acquire the effective channel between itself and all BSs by local CSI assumption described above. The acquired effective channels of each UE and radar can be represented by the multiplication of communication channel and signal space of receiving nodes. For example, the effective channel between *j*-th UE in *i*-th cell and *k*-th BS or and *p*-th radar are $U_{k}^{H}H_{k}^{\left[i,j\right]}$ and $U_{\left(p\right)}^{H}H_{p}^{\left(i,j\right)}$, respectively. Similarly, the effective channel between *p*-th radar and *k*-th BS is $U_{k}^{H}H_{k}^{p}$.

### 3.2. Transmit Beamforming

#### 3.2.1. Transmit Beamforming at Communication User Equipments

The *j*-th UE in the *i*-th cell uses the acquired effective channel from radars to compute interference matrix $\left(G^{\left(i,j\right)}\right)$, which is given by

(5) $$\begin{array}{c} G^{\left(i,j\right)}={\left[{\left(U_{\left(1\right)}^{H}H_{1}^{\left(i,j\right)}\right)}^{T},\cdots ,{\left(U_{\left(P\right)}^{H}H_{P}^{\left(i,j\right)}\right)}^{T}\right]}^{T}\in C^{P\times L}. \end{array}$$

Based on the interference matrix $G^{\left(i,j\right)}$, each UE independently computes its transmit beamforming vector to minimize the ISI from itself to radar system by utilizing singular value decomposition (SVD) of $G^{\left(i,j\right)}$ which is given by

(6) $$\begin{array}{c} G^{\left(i,j\right)}=\Omega^{\left(i,j\right)}\Sigma^{\left(i,j\right)}V^{(i,j)H}, \end{array}$$

where $\Sigma^{\left(i,j\right)}$ is P×L diagonal matrix with *L* singular values in diagonal components and zeros for other components, and $V^{\left(i,j\right)}$ denotes L×L right singular vector (RSV) matrix. Then, $\Sigma^{\left(i,j\right)}$ and $V^{\left(i,j\right)}$ can be represented by

(7) $$\begin{array}{c} \mathrm{diag}\left(\Sigma^{\left(i,j\right)}\right)=\left[\sigma_{1}^{\left(i,j\right)},\sigma_{2}^{\left(i,j\right)},\cdots \sigma_{L}^{\left(i,j\right)}\right]\;\mathrm{and}\;V^{\left(i,j\right)}=\left[v_{1}^{\left(i,j\right)},v_{2}^{\left(i,j\right)},\cdots v_{L}^{\left(i,j\right)}\right], \end{array}$$

respectively, where $\sigma_{1}^{\left(i,j\right)}>\sigma_{2}^{\left(i,j\right)}>\cdots >\sigma_{L}^{\left(i,j\right)}$ and $\sigma_{l}^{\left(i,j\right)}$ can be regarded as a amount of ISIs to radar system where the *j*-th UE in the *i*-th cell utilizes $v_{l}^{\left(i,j\right)}$ as the transmit beamforming vector. Hence, the *j*-th UE in the *i*-th cell takes $v_{L}^{\left(i,j\right)}$, i.e., the *L*-th RSV, as the transmit beamforming vector $w^{\left(i,j\right)}$ to minimize its ISI to radar system.

#### 3.2.2. Transmit Beamforming at Radar Transceivers

Similar with Section 3.2.1, the *p*-th radar calculates its interference matrix $\left(G_{p}\right)$ with the effective channel matrix received from BSs, which is given by

(8) $$\begin{array}{c} G_{p}={\left[{\left(U_{1}^{H}H_{1}^{p}\right)}^{T},\cdots ,{\left(U_{K}^{H}H_{K}^{p}\right)}^{T}\right]}^{T}\in C^{KS\times Y}. \end{array}$$

The $G_{p}$ indicates the ISI from the *p*-th radar to the communication system and then, radar performs the SVD as in Section 3.2.1 as follows

(9) $$\begin{array}{c} G_{p}=\Omega_{p}\Sigma_{p}V_{p}^{H}, \end{array}$$

where

(10) $$\begin{array}{c} V_{p}=\left[v_{p,1},\cdots ,v_{p,Y}\right]\in C^{Y\times Y}. \end{array}$$

Since Y>KS is assumed in our system model, $G_{p}$ becomes a fat matrix, i.e., $G_{p}$ has more columns than rows. Then, there exist Y−KS RSVs that can completely eliminate the ISI from the *p*-th radar to the communication system. Thus, the *p*-th radar uses these vectors to calculate the projection matrix $\left(W_{p}\right)$ to project the original radar signal while eliminating the ISI to communication system, which is given by

(11) $$\begin{array}{c} W_{p}=\left[v_{p,KS+1}\cdots ,v_{p,Y}\right]\times {\left[v_{p,KS+1}\cdots ,v_{p,Y}\right]}^{H}. \end{array}$$

After projection matrix is calculated, the transmit signal $\left({\hat{x}}_{p}\right)$ of the *p*-th radar is obtained by

(12) $$\begin{array}{c} {\hat{x}}_{p}=\frac{W_{p}x_{p}}{∥W_{p}x_{p}∥}, \end{array}$$

where $x_{p}$ is the original transmit signal of the *p*-th radar transceiver.

### 3.3. User Scheduling

As noted before, each BS in the communication system selects *S* UEs among *N* UEs in corresponding cell. The *j*-th UE in *i*-th cell calculates the amount of ICI from itself to the BSs in other cells, when they transmit uplink signal with the transmit beamforming vector $w^{\left(i,j\right)}$ determined in Section 3.2.1, which is given by

(13) $$\begin{array}{c} \eta^{\left[i,j\right]}=\sum_{k=1,k\neq i}^{K}U_{k}H_{k}^{\left[i,j\right]}w^{\left(i,j\right)}. \end{array}$$

After amount of ICI is measured, each UE feedbacks the calculated amount of ICI, $\eta^{\left[i,j\right]}$, to its corresponding BS. Then, all BSs selects S(≤N) UEs that generate minimum ICI to other BSs, where the index of the *s*-th scheduled UE in the *i*-th cell is given by

(14) $$\begin{array}{c} j_{s}^{\left[i\right]}=\underset{j\in N}{\mathrm{argmin}}\;\eta^{\left[i,j\right]},\;N=\left\{1,2,\cdots ,N\right\}\backslash \left\{j_{1}^{\left[i\right]},\cdots ,j_{s-1}^{\left[i\right]}\right\},\;1\leq s\leq S. \end{array}$$

## 4. Performance Metrics: Sum-Rate and Target Detection Probability

In this paper, we consider two performance metrics for validating the proposed OIAR technique: sum-rate for the communication system and the target detection probability for the radar system.

### 4.1. Sum-Rate for Communication Systems

The *s*-th selected UE in the *i*-th cell sends a communication signal with the determined transmit beamforming vector $w^{\left(i,j_{s}^{\left[i\right]}\right)}$, and then the received signal at the *i*-th BS can be expressed as

(15) $$\begin{array}{c} y_{i}=P_{u}\sum_{s=1}^{S}H_{i}^{\left[i,j_{s}^{\left[i\right]}\right]}w^{\left(i,j_{s}^{\left[i\right]}\right)}x^{\left[j_{s}^{\left[i\right]}\right]}+P_{r}^{*}\sum_{p=1}^{P}H_{i}^{p}{\hat{x}}_{p}+P_{u}\sum_{k=1,k\neq i}^{K}\sum_{s=1}^{S}H_{i}^{\left[k,j_{s}^{\left[k\right]}\right]}w^{\left(k,j_{s}^{\left[k\right]}\right)}x^{\left[j_{s}^{\left[k\right]}\right]}+z_{i}, \end{array}$$

where $x^{\left[k,j_{s}^{\left[k\right]}\right]}$, $P_{r}^{*}$, and $P_{u}$ indicate the signal transmitted by the *s*-th selected UE in the *k*-th cell, the received interference power from the radar at the BS, and the received UE signal power at the BS. The term $z_{i}\in C^{M\times 1}$ denotes the additive noise at each antenna of *i*-th BS, where each component follows the distribution of the complex Gaussian with zero-mean and unit-variance without loss of generality.

Then, the *i*-th BS performs the receive beamforming operation for the received signal as

(16) $$\begin{array}{c} r_{i}={\left[r_{i,1},\cdots ,r_{i,S}\right]}^{T}=F_{i}^{H}U_{i}^{H}y_{i}, \end{array}$$

where $F_{i}$ denotes a well-known zero-forcing equalizer to decode the received signal and it is defined as

(17) $$\begin{array}{c} F_{i}=\left[f_{i,1},\cdots ,f_{i,S}\right]={\left({\left[U_{i}^{H}H_{i}^{\left[i,j_{1}^{\left[i\right]}\right]}w^{\left(i,j_{1}^{\left[i\right]}\right)},\cdots ,U_{i}^{H}H_{i}^{\left[i,j_{S}^{\left[i\right]}\right]}w^{\left(i,j_{S}^{\left[i\right]}\right)}\right]}^{-1}\right)}^{H}.9 \end{array}$$

By exploiting (17), each decoded signal of *s*-th selected user in *i*-th cell in (16) can be rewritten as

(18) $$\begin{array}{c} r_{i,s}=P_{u}x^{\left[i,j_{s}^{\left[i\right]}\right]}+P_{r}^{*}\sum_{p=1}^{P}f_{i,s}^{H}U_{i}^{H}H_{i}^{p}{\hat{x}}_{p}+P_{u}\sum_{k=1,k\neq i}^{K}\sum_{l=1}^{S}f_{i,s}^{H}U_{i}^{H}H_{i}^{\left[k,l\right]}W_{s}^{\left(k,l\right)}x^{\left[k,l\right]}+f_{i,s}^{H}U_{i}^{H}z_{i}. \end{array}$$

Then, the sum-rate for communication systems is given by

(19) $$\begin{array}{c} R=\sum_{i=1}^{K}\sum_{s=1}^{S}\log_{2}\left(1+\frac{{\mathrm{SNR}}_{u}}{∥f_{i,s}∥+{\mathrm{SNR}}_{u}I_{i,s}+{\mathrm{INR}}_{r}I_{i,s}^{R}}\right), \end{array}$$

where ${\mathrm{SNR}}_{u}$ and ${\mathrm{INR}}_{r}$ denote the received SNR from UE and the received INR from radar at BS. Moreover, $I_{i,s}$, and $I_{i,s}^{R}$ represent the ICI from UEs that does not belong to *i*-th cells and the ISI from radars, which is given by

(20) $$\begin{array}{c} I_{i,s}=\sum_{k=1,k\neq i}\sum_{s=1}^{S}{\left|f_{i,s}^{H}U_{i}^{H}H_{i}^{\left[k,j_{s}^{\left[i\right]}\right]}w^{\left(k,j_{s}^{\left[i\right]}\right)}\right|}^{2}\mathrm{and}\;I_{i,s}^{R}=\sum_{p=1}^{P}{\left|f_{i,s}^{H}U_{i}^{H}H_{i}^{p}{\hat{x}}_{p}\right|}^{2}. \end{array}$$

### 4.2. Detection Probability for Radar Systems

Since only the LOS channel is assumed between each radar and its target of interest, the signal of the radar does not experience distortion caused by multipath fading. After receive beamforming, the received signal at the *p*-th radar consists of the signal reflected from the target of interest and the ISI from the UEs in the communication system, which can be expressed as

(21) $$\begin{array}{cc} y_{\left(p\right)}= & P_{r}a_{p}^{H}{\hat{x}}_{p}+P_{u}^{*}a_{p}^{H}\sum_{k=1}^{K}\sum_{s=1}^{S}H_{p}^{\left(k,j_{s}^{\left[k\right]}\right)}w^{\left(k,j_{s}^{\left[k\right]}\right)}x^{\left[k,j_{s}^{\left[k\right]}\right]}+z_{\left(p\right)}, \end{array}$$

where $z_{\left(p\right)}$ denotes the additive noise at the *p*-th radar transceiver, which becomes the complex Gaussian random variable with zero-mean and unit-variance. Moreover, $P_{r}$ and $P_{u}^{*}$ represent the received radar signal power at the radar and the received interference power from the UE at the radar. As explained in (12), each radar forms the transmit radar signal by multiplying the projection matrix and original radar signal so that the received radar signals at all BS are nulled out.

To calculate the target detection probability at radar transceivers, we adopt following Equation 12,

(22) $$\begin{array}{c} P_{D}^{p}=1-F_{\chi_{2}^{2}\left(\rho_{p}\right)}\left(F_{\chi_{2}^{2}}^{-1}\left(1-P_{FA}\right)\right), \end{array}$$

where $P_{FA}$ is a desired probability of false alarm, $F_{\chi_{2}^{2}}^{-1}$ is the inverse central chi-squared cumulative distribution function with two degrees of freedom, and $F_{\chi_{2}^{2}\left(\rho_{p}\right)}$ is the noncentral chi-squared cumulative distribution function with two degrees of freedom with noncentrality parameter $\rho_{p}$. The noncentrality parameter, $\rho_{p}$ can be calculated by covariance between transmit signal and received signal as [22]

(23) $$\begin{array}{c} \rho_{p}=\frac{{\mathrm{SNR}}_{r}{\left|a_{p}^{H}W_{p}x_{p}x_{p}^{H}W_{p}^{H}a_{p}\right|}^{2}}{1+{\mathrm{INR}}_{u}I_{\left(p\right)}},\;I_{\left(p\right)}=\sum_{k=1}^{K}\sum_{s=1}^{S}{\left|a_{p}^{H}H_{p}^{\left(k,j_{s}^{\left[k\right]}\right)}w^{\left[k,j_{s}^{\left[k\right]}\right]}{\hat{x}}_{p}^{H}a_{p}\right|}^{2}, \end{array}$$

where ${\mathrm{SNR}}_{r}$ and ${\mathrm{INR}}_{u}$ denote received SNR at radar and received INR at radar from UE, respectively. $I_{\left(p\right)}$ represents the amount of ISI from the UEs in the communication system to the *p*-th radar.

## 5. Numerical Results

In this section, we show simulation results that demonstrate performances of the proposed OIAR technique and compare the sum-rate and the target detection probability with the conventional NSP techniques [22]. Since only a single radar system is considered in [22], we apply a basic OIA technique at communication system for fair comparison. In NSP with OIA technique, each radar system selects the best BS that has the minimum performance degradation as in [33]. It is worth noting that the proposed technique operates with a fully distributed manner, which implies that each radar transceiver, each UE, and each BS do not exchange control signals for coordinate protocols to each other. The system parameters for simulations are summarized as follows: K=3, M=4, N=20, L=2, Y=32, $\theta_{p}=0^{\circ }\;\forall p$, $P_{FA}=10^{-5}$.

**Figure 3:**
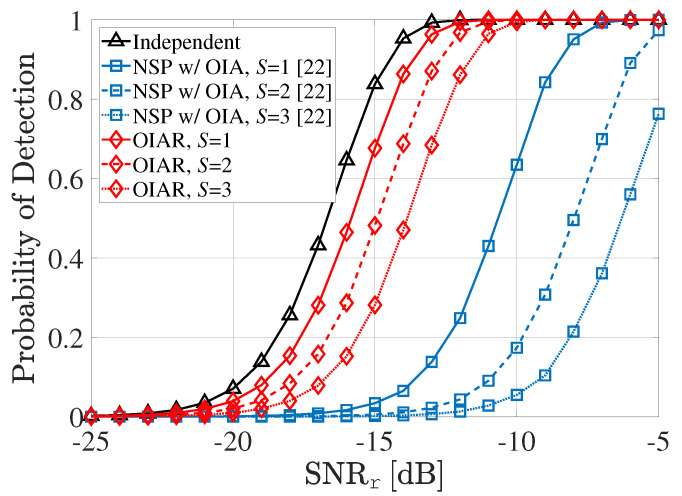
Radar probability of detection according to ${\mathrm{SNR}}_{r}$ with different *S*.

In Figure 3, the target detection probability of radar systems is shown according to ${\mathrm{SNR}}_{r}$ when ${\mathrm{INR}}_{u}=-15\;\mathrm{dB},P=1$ with different *S* (i.e., number of selected UEs per cell). Since the NSP with OIA technique operates only with projection of radar signal into null space of the best BS and the interference from communication system to radar does not considered, the target detection probability of the proposed OIAR significantly outperforms the conventional NSP with OIA. Even though OIAR cant not completely eliminate the ISI from UEs as *S* increases, it is shown that its degradation is much less than the conventional NSP with OIA technique. As a reference, we consider the independent case that the radar system operates without any interference from communication systems, which is illustrated with a black line, since two systems use different spectrum bands.

**Figure 4:**
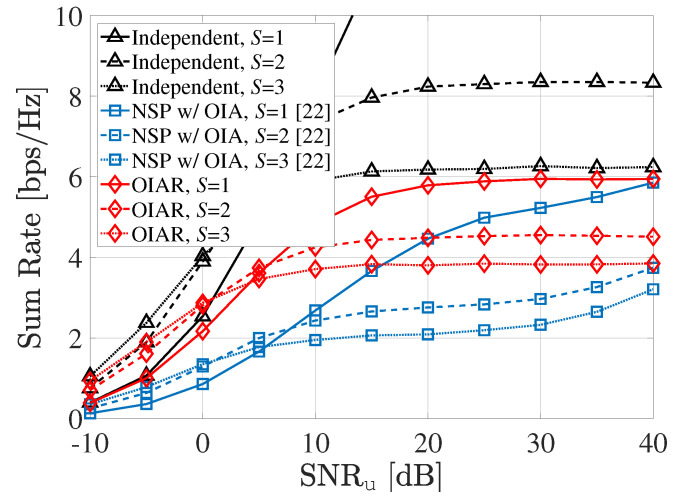
Sum-rate of communication system according to ${\mathrm{SNR}}_{u}$ with different *S*.

In Figure 4, the sum-rate of the communication system is shown according to ${\mathrm{SNR}}_{u}$ when ${\mathrm{INR}}_{r}=50\;\mathrm{dB},P=1$ with different *S* (i.e., number of selected UEs per cell). The sum-rate of the proposed OIAR technique outperforms the conventional NSP with OIA technique as well. The transmit beamforming vector of UEs with OIAR technique are designed for minimizing the ISI to radar system, and thus the sum-rate becomes obviously lower than the independent case.

**Figure 5:**
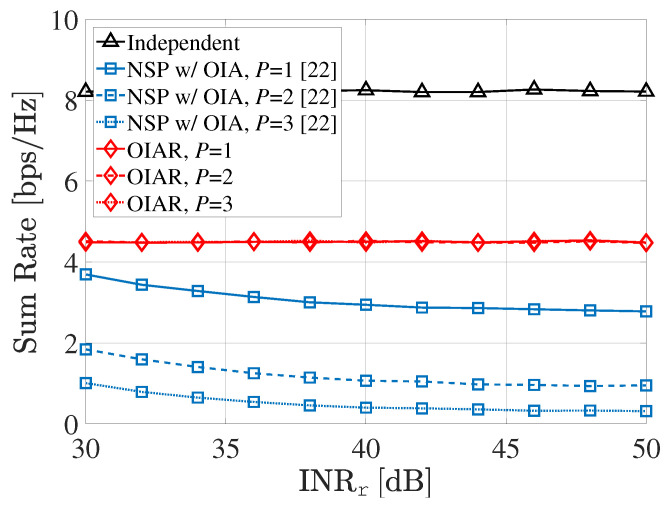
Sum-rate of communication system according to ${\mathrm{SNR}}_{r}$ with different *P*.

Figure 5 shows the sum-rate of communication systems according to ${\mathrm{INR}}_{r}$ with different *P* (i.e., number of radars) where $S=2,{\mathrm{SNR}}_{u}=20\;\mathrm{dB}$. In Figure 5, the sum-rate of the proposed OIAR technique is not degraded even though the received INR from the radar system at BSs increases because the ISI from the radar to the communication system is completely eliminated by the large number of radar antennas, while the sum-rate of NSP with OIA technique becomes degraded as ${\mathrm{INR}}_{r}$ increases.

**Figure 6:**
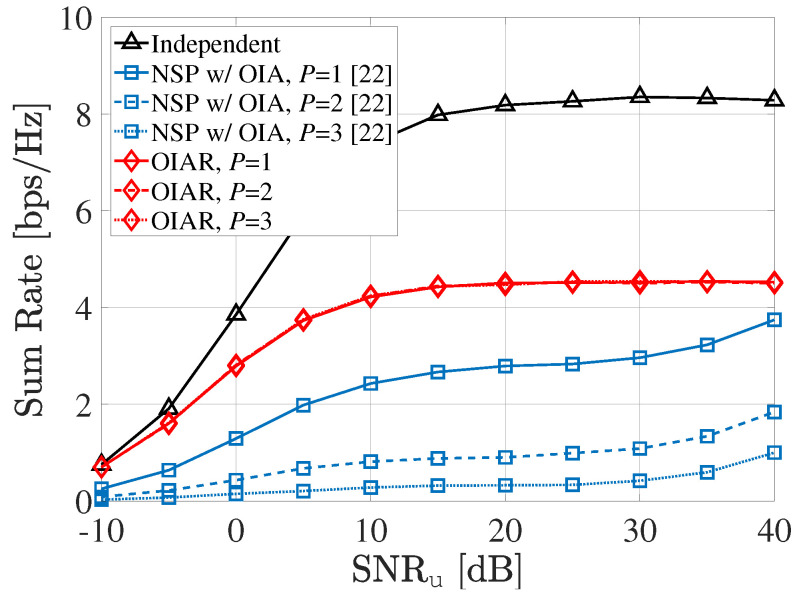
Sum-rate of communication system according to ${\mathrm{SNR}}_{u}$ with different *P*.

The sum-rate performance of communication system according to ${\mathrm{SNR}}_{u}$ with different *P* (i.e., number of radars) is shown in Figure 6, where $S=2,{\mathrm{INR}}_{r}=50\;\mathrm{dB}$. Since Y>KS is assumed by the large number of radar assumption [42], radars could not affect any ISI to BSs because transmit signal of radars are in the null space of effective interference channel between radar and BSs. Thus, it is shown that sum-rate performance of proposed OIAR is not changed by the number of radars, *P*, unlike conventional NSP technique.

**Figure 7:**
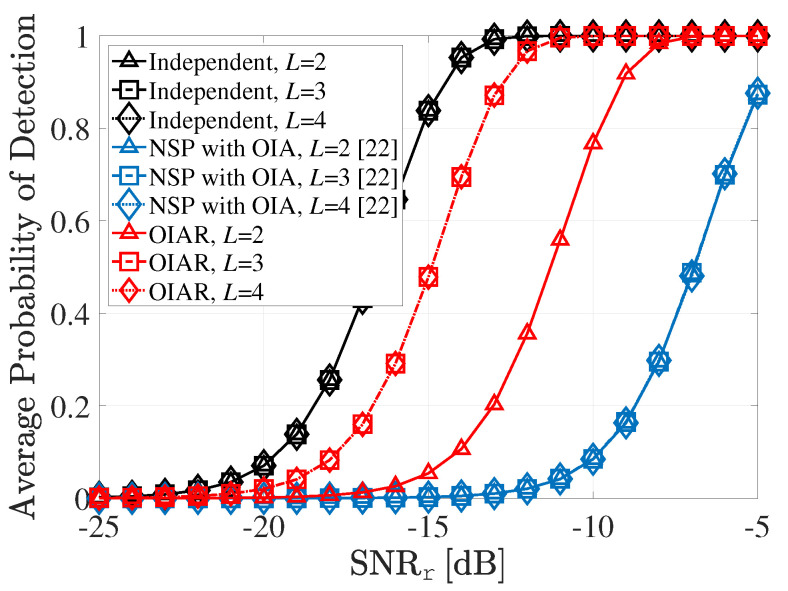
Average probability of detection performance according to ${\mathrm{SNR}}_{r}$ with different *L*.

To show the effect of number of antennas of UE, average probability detection performance according to ${\mathrm{SNR}}_{r}$ with different *L* (i.e., number of UE antennas) is shown in Figure 7, where M=8,P=2,S=2 and other parameters are same with former simulations. Since transmit beamforming vector of UEs are designed by *L*-th right singular vector of interference matrix from UE to radars, if L>P is satisfied, then the ISI from UEs to radars are completely eliminated. Hence, there is performance degradation in OIAR where L=2 because there is residual ISI from UEs to radars, since L>P is not satisfied. However, even though the performance of OIAR is degraded, it still achieves better performance than conventional NSP technique.

**Figure 8:**
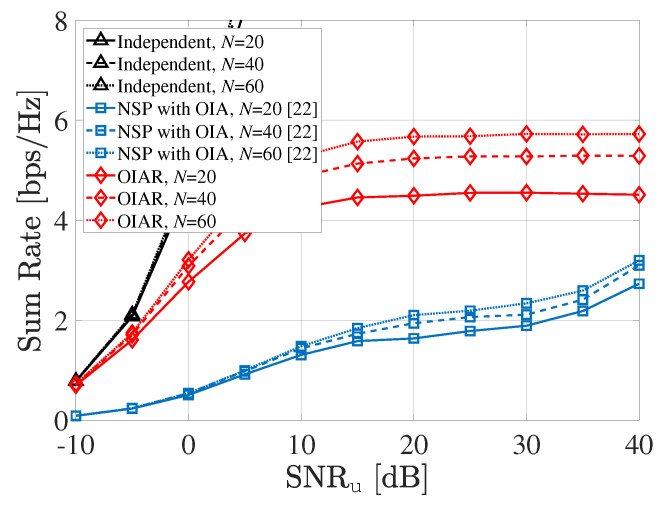
Sum-rate of communication system according to ${\mathrm{SNR}}_{u}$ with different *N*.

In Figure 8, the sum-rate performance of communication system is illustrated according to ${\mathrm{SNR}}_{u}$ with different *N* (i.e., number of UEs per cell), where $P=2,S=3,L=3,{\mathrm{INR}}_{r}=50\;\mathrm{dB}$ and other parameters are same with common parameters mentioned in first paragraph of this chapter. As more UEs are exist in a cell, there is more selection diversity to give opportunity to choose better UEs than when *N* is small. Because of that selection diversity, both OIAR and NSP technique achieves better performance with large number of *N* than when *N* is small. However, since NSP could not manage ISI from radar properly in our considered scenario, its sum rate performance is poor than proposed OIAR technique.

## 6. Conclusions

In this paper, we proposed an opportunistic interference alignment with radar (OIAR) technique for spectrum sharing between radar and uplink communication systems, where IA based radar signal projection and transmit beamforming of user equipment (UE) in communication systems are performed. With the radar signal projection, inter-system interference (ISI) from the radar system to base stations (BSs) in communication systems is completely eliminated, while the ISI from UEs to radar transceivers can be effectively mitigated. Through extensive simulations, it is shown that the proposed OIAR technique significantly outperforms the conventional null-space projection technique.
